# Completeness of hepatitis, brucellosis, syphilis, measles and HIV/AIDS surveillance in Izmir, Turkey

**DOI:** 10.1186/1471-2458-10-71

**Published:** 2010-02-17

**Authors:** Raika Durusoy, Ali O Karababa

**Affiliations:** 1 Department of Public Health, Ege University Medical School, Izmir, Turkey

## Abstract

**Background:**

According to the surveillance system in Turkey, most diseases are notified only by clinicians, without involving laboratory notification. It is assumed that a considerable inadequacy in notifications exists; however, this has not been quantified by any researcher. Our aim was to evaluate the completeness of communicable disease surveillance in the province of Izmir, Turkey for the year of 2003 by means of estimating the incidences of diseases.

**Methods:**

Data on positive laboratory results for the notifiable and serologically detectable diseases hepatitis A, B, C, brucellosis, syphilis, measles and HIV detected in 2003 in Izmir (population 3.5 million) were collected from serology laboratories according to WHO surveillance standards and compared to the notifications received by the Provincial Health Directorate. Data were checked for duplicates and matched. Incidences were estimated with the capture-recapture method. Sensitivities of both notifications and laboratory data were calculated according to these estimates.

**Results:**

Among laboratories performing serologic tests (n = 158) in Izmir, 84.2% accepted to participate, from which 23,515 positive results were collected. Following the elimination of duplicate results as well as of cases residing outside of Izmir, the total number was 11,402. The total number of notifications was 1802. Notification rates of cases found in laboratories were 31.6% for hepatitis A, 12.1% for acute hepatitis B, 31.8% for brucellosis, 25.9% for syphilis and 100% for HIV confirmation.

**Conclusions:**

It was discovered that for hepatitis A, B, C, brucellosis and syphilis, there is a considerable under-notification by clinicians and that laboratory data has the potential of contributing greatly to their surveillance. The inclusion of laboratories in the surveillance system of these diseases could help to achieve completeness of reporting.

## Background

Laboratory notification has become an integral part in the surveillance system of many countries as a result of advances in laboratory diagnosis. According to the communicable disease surveillance system in Turkey, most diseases are expected to be notified only by clinicians, without involving laboratory notification. This route leads to significant under-estimation of disease burden. According to a study carried out on hepatitis A cases in a paediatric state hospital in Izmir in 1999, only 30.2% of the 351 cases with a positive IgM antiHAV laboratory result were reported [1]. Although these data existed in the laboratory of the hospital, they were not sent to the local health authority as there was no such chain of notification defined and as there is a considerable underreporting by clinicians.

The objectives of this study were:

1. To assess completeness of surveillance of some notifiable diseases by combining laboratory data and notifications.

2. To quantify under-notification using capture-recapture.

3. To evaluate the contribution of laboratory data to notifications in order to give recommendations on how laboratory data can best improve the surveillance system in Turkey.

## Methods

### Scope

According to the communicable disease surveillance system in operation during the study, there were 39 notifiable diseases [2]. For practicality in data collection, diseases having serological diagnostic procedures according to WHO surveillance standards [3] were selected: hepatitis A, B, C, brucellosis, syphilis, measles and HIV/AIDS. The results of the serological parameters shown in Table 1 were collected as data.

**Table 1:** Serological parameters used in the study for data collection

Disease	**WHO serological criteria **[3]	Additional criteria and relevance
Hepatitis A	IgM anti-HAV positive (acute)	-
Hepatitis B	HBsAg positive (unspecified) or IgM anti-HBc positive (acute)	Total anti-HBc positive when IgM Anti-HBc not tested HBV-DNA positive
Hepatitis C	anti-HCV positive (acute and unspecified combined)	HCV-confirmation positive HCV-RNA positive, showing also infectivity
Brucellosis^a^	Brucella agglutination titre (e.g., standard tube agglutination > 160) or ELISA (IgA, IgG, IgM), 2-ME (confirmed)	Rose-Bengal test, due to its use as a screening test (probable)
Syphilis	RPR or VDRL confirmed by TPHA or FTA	-
Measles^b^	Specific IgM antibodies	-
HIV infection	HIV positive serology (ELISA), confirmation	HIV-RNA positive

^a ^For brucellosis, complement fixation, FAT and RIA for detecting antilipopolysaccharide antibodies also exist among WHO criteria but as these tests were not being performed in any laboratory in Izmir, they are not used as criteria.

^b ^For measles, "at least a fourfold increase in antibody titre and virus isolation" is also stated among WHO criteria. But as it would not be practical to follow-up the first criteria and as virus isolation was not performed in any laboratory in Izmir, these criteria were not used.

All the province of Izmir (nine urban and 19 rural districts) with its 3.5 million inhabitants was covered in this research since inhabitants of a district could be diagnosed in other districts with more numerous and sophisticated medical facilities. The city of Izmir, administrative centre of the province, is the third largest metropolitan area of Turkey and with two university hospitals and six other public tertiary care hospitals, there would be little chance for cases to be diagnosed elsewhere. By procedure, notifications from all districts are sent to Izmir Provincial Health Directorate, enabling comparison at a provincial level.

In order to contact all serology laboratories in the province, the list of public and private facilities providing laboratory services was obtained from the provincial health directorate. All these facilities were visited (n = 190) in search for laboratories using serological diagnostic procedures. Facilities not applying serology (n = 21) and facilities which were no longer in operation (n = 11) were excluded. Facilities measuring at least one of the serological parameters stated in Table 1 were the target group of the study (n = 158).

### Data collection and analysis

Data on cases that were diagnosed in the year 2003 were collected between 21 January 2003 and 25 March 2005. A total of 133 serology laboratories (84.2%) agreed to participate in the study. Table 2 shows the distribution of facilities performing serological tests in Izmir according to their agreeance to participate, their locations and their types.

**Table 2:** Locations, type and acceptance status of facilities performing serological tests in Izmir province

	Accepted		Rejected		Total	
	**n**	**%**	**n**	**%**	**n**	**% among total**

Total in the urban area	92	80.0	23	20.0	115	72.8
Total public	26	92.9	2	7.1	28	17.7
Hospital	19	95.0	1	5.0	20	12.7
Blood donation centre^a^	4	80.0	1	20.0	5	3.2
Laboratory	3	100.0	-		3	1.9
Total private	66	75.9	21	24.1	87	55.1
Hospital	9	90.0	1	10.0	10	6.3
Laboratory	57	74.0	20	26.0	77	48.7
Total out of the urban area	41	95.3	2	4.7	43	27.2
Total public	17	94.4	1	5.6	18	11.4
Hospital	15	100.0	-		15	9.5
Blood donation centre	1	100.0	-		1	0.6
Laboratory	1	50.0	1	50.0	2	1.3
Private laboratory	24	96.0	1	4.0	25	15.8
Total	133	84.2	25	15.8	158	100.0

^a ^The four cooperating centres are part of hospitals but are classified as separate facilities due to difference in their test procedures.

Data concerning cases having positive serology results in 2003 were collected from participating laboratories. Most of these data were actively collected on consecutive visits: Cases were found by scanning records, usually on paper/laboratory notebooks and information about their identities was traced among administrative records, usually in a computer system. In some laboratories where results were not retained and there were few cases identified, we left case report forms for the laboratory to complete. Facilities were also asked to send electronic notifications to the researcher. Some laboratories accepted and sent results electronically. But these facilities were also visited for data collection, as some of them did not send electronic notifications although they had agreed to.

Laboratory results and notifications were entered into the same database. The database included one row for each laboratory test day for cases found positive. Data were matched based on full name, and for similar names additional data including date of birth, sex, father's name, address, phone number, date of testing and diagnosis were used to match cases. Duplicates in multiple laboratories were classified according to the laboratory which first diagnosed them. For HIV/AIDS, both data sources did not include names but were coded with a standard process relying on surname, name, father's name and birth year. Cases were compared according to these standard codes and only a 100% match on the code was used to link records.

As notifications to Izmir Provincial Health Directorate consisted only of cases residing in Izmir, laboratory - diagnosed cases not residing in Izmir also needed to be removed from the analysis. To do this, cases with missing addresses were classified as cases from Izmir if they were found positive out of the urban area or in secondary care institutions inside the metropolitan area (6.5% of cases), since cases with a referral sent from other provinces to Izmir are cared for in tertiary care institutions. Cases with a missing address in tertiary care (25.1% of cases) were assigned randomly as Izmir or non-Izmir cases, proportionate to the number of non-Izmir cases found in each institution among cases with known addresses. Laboratory diagnosis rates and reported rates were calculated using numbers of cases found in each source and 2003 mid-year population of Izmir province.

### Application of the capture-recapture method

Capture-recapture is a method used to estimate the number of individuals in a population using population-based data from two or more independent, but overlapping sources [4]. Its name deriving from its first usage by zoologists, it has been used in human populations since 1949 [5,6]. In recent years, it has been used by epidemiologists to estimate prevalence or incidence of several diseases, to evaluate completeness of different notifications and to estimate coverage of cancer registries [4,7-11]. The underlying reason for its usage is the insufficiency of disease surveillance systems in covering all cases and its appropriateness, as a method, in correcting this insufficiency [12,13].

Incidence rates were calculated using Izmir's mid-year population in 2003. Incidences and sensitivity of notifications were estimated with capture-recapture method using two data sources: cases found positive in laboratories and cases notified. Estimates were calculated with Chapman's formula developed for two-list capture-recapture [6] and Chao's lower bound estimator using frequency data adapted for a two-source capture-recapture [14]. Both formulas are given below.

Chapman's formula:

Where *L*_1 _is the number of cases in the first source (laboratory), *L*_2 _is the number of those present in the second source (notifications), and *d *is the number of cases present on both lists.

Chao's lower bound estimator:

Where *f*_1 _is the frequency of those identified by exactly one source (equal to [*L*_1_+*L*_2_-2*d*] in Chapman's formula), and *f*_2 _the frequency of identifications by exactly two sources (equal to [*d*]).

### Ethical considerations

A written permission was received from the Ministry of Health. Ethical committee approval was obtained from a local tertiary care hospital providing graduate medical education.

The anonymity and security of identities of cases was of major importance in this study. The confidentiality of data was guaranteed to participating facilities and to TÜBİTAK; the institution granting this research. Data were collected by only one researcher who entered and stored it in a password-protected database.

## Results

Among laboratories performing serological tests, 93.5% public (n = 46) and 80.4% private (n = 112) facilities accepted to participate in the study (Table 2).

Data were collected thoroughly from 77.4% (n = 103) of the participating institutions for the year 2003. In 5.3% of the facilities (n = 7), data could not be obtained due to the lack of record-keeping. In 7.5% (n = 10) data on some months were missing, in 6.8% (n = 9) data for some months could not be accessed due to unsolvable technical problems, and in 3.0% (n = 4) the laboratories were closed in the year 2003 so data were available only for the months they were open.

Internet connection was available in 42.1% (n = 56) of participating facilities, among which 44.6% (n = 25) accepted to send electronic notifications to the researcher. Only four facilities sent electronic notifications. Another facility sent the addresses of cases electronically. No positive case was found in four of the 20 facilities which did not send electronic notifications.

In total, 628 visits were made to participating facilities and data concerning 19,458 positive results were collected. With the addition of 4057 (17.3%) positive results sent electronically, the total number of positive results collected during this study amounted to 23,515. Among positive results, 93.2% were diagnosed in public versus 6.8% in private institutions and 89.2% were diagnosed in hospitals, 7.1% in laboratories and 3.7% in blood donation centres. Among all positive results, 80.4% were diagnosed in only 11 public institutions (8.6% of facilities) of which 68.4% consisted of positive results for hepatitis B. In 20.9% (n = 27) of the facilities, no positive result was found and all of these were private laboratories.

After the elimination of duplicates, 23515 positive results corresponded to 17319 cases. Among these, 11402 cases were classified as cases from Izmir. For the same period, 1802 cases were notified to the directorate for the same diseases, 488 of them common in total. The distribution and quantity of cases found in laboratories and the notifications are displayed in Table 3 based on different levels of parameters. Notification rates of cases found in laboratories and reported rates are shown in the same table. Notification rates of cases found in laboratories were the highest for HIV (100%), about one third for hepatitis A, brucellosis and syphilis, 12% for acute hepatitis B and 1-4% for hepatitis C. Explanations for the use of different levels of parameters are discussed below in the paragraphs related to each disease. Table 4 shows capture-recapture estimates of the total numbers of cases, sensitivities of each source and incidence estimates.

**Table 3:** Numbers of cases of each disease and notification rates in Izmir, 2003 (N = 3,506,672)

	No. of records in the data sources				Rates according to data sources (per 100,000)		
	Lab diagnoses (a)	Notifications to Health Directorate (b)	Matched records (c)	Proportion of lab diagnoses notified % (c/a)	Lab diagnosis rate (a/N)	Notification rate (b/N)	Combined rate ([a+b-c]/N)
Hepatitis A	560	587	177	31.6	15.97	16.74	27.66
Hepatitis B							
WHO criteria	10285	225	157	1.5			
IgM anti-HBc	380	225	46	12.1	10.84	6.42	15.94
Hepatitis C							
WHO criteria	148	89	2	1.4			
Hepatitis C total	2271	89	75	3.3			
Dialysis & gastroenterology cases omitted	1757	89	75	4.3	50.10	2.54	50.50
Brucellosis							
WHO criteria	151	116	48	31.8	4.31	3.31	6.25
Agglutination ≤ 1/80	307	116	65	21.2	8.75	3.31	10.21
Syphilis							
WHO criteria	201	86	52	25.9	5.73	2.45	6.70
notified lab cases not meeting WHO criteria included	217	86	68	31.3	6.19	2.45	6.70
Measles	5^a^	272	0	0	0.14	7.76	7.87
HIV							
Confirmation or RNA positive	23	15	6	26.1	-	0.17	-
Confirmation or RNA positive, previous years' notifications included	23	154^b^	23^b^	100.0	-	-	-
Total	11402	1802	488	4.3	-	-	-

^a ^One of the positive cases is due to vaccination

^b ^Notifications of previous years included

**Table 4:** Capture-recapture estimates of total numbers of cases, sensitivities of each source, incidence estimates and 95% confidence intervals

	Chapman's formula				Chao's lower bound estimator			
	Total number of cases in population	Sensitivity lab %	Sensitivity notifications %	Incidence (per 100,000)	Total number of cases in population	Sensitivity lab%	Sensitivity notifications %	Incidence (per 100,000)
Hepatitis A	1852 (1665-2040)	30 (28-34)	32 (29-35)	52.8 (47.5-58.2)	1858 (1669-2047)	30 (27-34)	32 (29-35)	53.0 (47.6-58.4)
Hepatitis B								
IgM anti-HBc	1831 (1399-2263)	21 (17-27)	12 (10-16)	52.2 (39.9-64.5)	1989 (1502-2477)	19 (15-25)	11 (9-15)	56.7 (42.8-70.6)
Hepatitis C								
dialysis & gastroenterology omitted	2081 (1901-2260)	84 (78-92)	4 (4-5)	59.3 (54.2-64.5)	11359 (8997-13721)	15 (13-20)	1 (1-1)	323.9 (256.6-391.3)
Brucellosis								
WHO criteria	362 (299-425)	42 (36-51)	32 (27-39)	10.3 (8.5-12.1)	371 (304-439)	41 (34-50)	31 (26-38)	10.6 (8.7-12.5)
Agglutination < 1/80	545 (468-622)	56 (49-66)	21 (19-25)	15.5 (13.4-17.7)	688 (572-804)	45 (38-54)	17 (14-20)	19.6 (16.3-22.9)
Syphilis								
WHO criteria	331 (283-378)	61 (53-71)	26 (23-30)	9.4 (8.1-10.8)	396 (327-465)	51 (43-61)	22 (19-26)	11.3 (9.3-13.3)
notified lab cases not meeting WHO criteria included	274 (250-298)	79 73-87)	31 (29-34)	7.8 (7.1-8.5)	338 (293-382)	64 (57-74)	25 (23-29)	9.6 (8.4-10.9)

For **hepatitis B**, the number of cases meeting WHO criteria was 10285, compared with 225 notifications. As HBsAg, is positive in carrier or chronic cases, and as Turkey is an intermediate-endemic country for Hepatitis B [15], a second analysis was performed with IgM anti-HBc positive cases only, yielding 380 cases diagnosed in laboratories and an incidence estimate (Tables 3, 4).

Among the **hepatitis C **cases discovered in laboratories in Izmir, few met WHO criteria (n = 148), with only two of them present among notifications. A further analysis was conducted including all anti-HCV positive cases (Tables 3, 4). At least 14.1% (n = 320) of cases found in laboratories were dialysis patients, 14.1% (n = 320) were found positive during blood donor testing, and 8.5%(n = 194) were admitted to a gastroenterology unit. The admitting unit was not known in 22.8% of cases. The dialysis patients in particular were regularly tested serologically at two-month intervals. Of the anti-HCV positive cases found during blood donor testing, 18.8% (n = 60) were notified and constituted the majority of notifications (n = 89). As none of the dialysis/gastroenterology cases were notified, they were omitted and a third analysis was conducted with the remaining 1757 cases to prevent a much higher capture-recapture estimate due to possible negative dependence.

Among **brucellosis **cases, 25.1% (n = 151) met WHO criteria. Some laboratories had used cut-off 1/80 for agglutination, which was also preferred by some authors [16], so a second analysis was performed using this cut-off, which included more of the notified cases in the analysis (Tables 3, 4).

Among **syphilis **notifications, 79.1% (n = 68) were present on the laboratory list but only 52 of them conformed to WHO criteria.

As none of the five **measles **cases discovered in laboratories were notified, a capture-recapture estimate was not calculated. Of the 272 cases notified, 76.5% were diagnosed in primary health centres where there is no laboratory testing for measles.

For **HIV/AIDS**, there were 206 positive cases detected in laboratories according to WHO criteria of which 54 were notified. As a serological parameter showing acute/recent infection is lacking for HIV/AIDS -as well as for hepatitis C-cases found positive in laboratories might be cases that were diagnosed in previous years. It was possible to check previous years' notifications for HIV/AIDS, but not for hepatitis C. In total 168 cases were estimated to be from Izmir of which 10 of them were notified in 2003 and a further 24 notified in previous years. As many anti-HIV affirmations could be cases of false-positive [17], and as only confirmed cases are notified as a rule in Izmir, only the results of confirmed cases (confirmation or RNA positive) are shown in Table 4, giving a notification rate of 100% when compared with all (2003 or earlier) HIV/AIDS notifications. Since 100% of cases were notified, a capture-recapture estimate was not calculated.

## Discussion

This study provides evidence of considerable underreporting of hepatitis, brucellosis and syphilis in Izmir, Turkey. The surveillance system captured less than 1/3 of cases diagnosed in laboratories and the potential contribution of laboratory data to their surveillance was significant. Official rates of disease were less than 1/3 to 1/8 of incidences estimated. Surveillance of HIV/AIDS was satisfactory with 100% of confirmed cases notified. As few facilities actually sent electronic notifications to the researcher, it might be premature to implement electronic notifications in the surveillance system.

### Strengths and limitations of the study

This study has the strength of comparing the contribution of laboratory data to notifications in the surveillance of different diseases in Izmir. This is the first comprehensive study on the evaluation of completeness of communicable disease surveillance in Turkey through collection of data from a source independent of the ministry's official notifications.

As Izmir is a referral centre for Aegean Region, the design was convenient for covering cases from Izmir. This study could not have been conducted in another province in the region since there would have been many cases applying to facilities in Izmir for secondary and tertiary care, reducing coverage of local cases.

Data were collected thoroughly in 77.4% of participating facilities. As for the remainder, inaccessibility to data for the first 3.5 months of 2003 of a tertiary care hospital (one of the six facilities with > 1000 positive results) and lack of data from the virology department of a public health laboratory of the ministry (a facility contributing with 97 positive results for brucellosis and syphilis with its bacteriology department) are considered as an important limitation of the study. Another limitation is the missing residence for one fourth of cases in tertiary care, which could have caused some misclassification when eliminating cases out of Izmir.

Among the non-participating facilities, lack of hepatitis B and C cases from one public blood donation centre is considered to be significant. The other non-participating facilities are not considered to cause much limitation since most of them are small-scale private laboratories which would have found only 1-5 positive results throughout the year or no positive result at all.

Another limitation is the use of different serological test procedures with different sensitivities and specificities for the same parameter in different laboratories, possibly leading to some false positive and false negative results which cannot be controlled due to the lack of clinical data in this study. This has also caused carrier or chronic cases to be on the laboratory list. The surveillance system in operation in 2003 did not have any case definitions for notifications, so carrier cases were also found among notifications, along with probable cases.

### Accuracy of capture-recapture analysis

There are four main assumptions that should be evaluated before implementing a capture-recapture analysis [6]. The province of Izmir can be considered a closed population. However; during the course of the research which covered the whole of the year 2003, there might have been some in- or out- migration from the city, which would have falsified to a small degree the assumption that the population is a closed one.

Theoretically, the two lists could be positively dependent, since the notification probability of a case diagnosed in laboratory could be greater. Chronic cases or cases with severe disease are more likely to be notified [18]. Positive dependence would result in the calculation of a smaller estimate, thus estimation of a higher completeness of notifications [6,9,18]. Inaccessibility to the first 3.5 months' data of a tertiary care hospital might have created negative dependence since the cases notified from that hospital in that period do not have the possibility of being on the laboratory list, if not also diagnosed elsewhere. This problem is not anticipated for the virology department of the public health laboratory since they do not notify any disease except HIV/AIDS. However; some of the few cases notified by primary health centres might have been diagnosed in this laboratory. Overall, when this study was conducted, laboratory confirmation was not required for notifying a case, and as the results show, notifications and laboratory reports are almost independent samples, combined with the considerable underreporting of laboratory cases. For hepatitis C, some of the cases found in labs could have been cases from previous years, leading to some negative dependence and thus a considerably high estimate.

As for the assumption on equal catchability, this might have been affected by the lack of data in some participating facilities and laboratories that did not accept to participate in the study, both of which are discussed above.

Capture-recapture results according to both methods are similar when the number of cases appearing on both lists is relatively higher. When different, estimates calculated according to Chapman's formula can be considered more cautiously, while Chao's method might be more realistic if there is an underestimation due to some positive dependence.

### Possible mechanisms and implications

This study has showed that there is a considerable underreporting of infectious diseases in Izmir. A similar underreporting might be expected for the whole of Turkey, with official reported rates 9.64 and 7.3 per 100,000 for hepatitis A and B [19], much lower than incidences estimated for Izmir which is considered as one of the most developed provinces where incidences might be expected to be lower. The reported incidence of 20.30 per 100,000 for brucellosis in Turkey might also need caution in interpretation [19], considering its underreporting rate in Izmir.

This study has also showed that laboratory data can make an important contribution to the surveillance system of these diseases classified as Group A in the new system [20]. According to the new communicable disease surveillance system introduced as of the beginning of 2005, most of the notifiable diseases are classified as Group A which are notified by clinicians as in the previous system and laboratories are not involved in this passive surveillance [21]. Only a limited number of pathogens classified as Group D are reported by laboratories (Shigella, Salmonella, EHEC, Campylobacter, Listeria monocytogenes, Entamoeba histolytica, Cryptosporidium, Giardia intestinalis and Chlamydia trachomatis). The only changes in the system for group A diseases are the introduction of standard case definitions and the classification of cases as confirmed/probable. The reason why a considerable proportion of cases notified have not been diagnosed in laboratories could be the lack of case definitions in the previous system.

Among types of facilities, hospitals were significant with the diagnostic rate of 89% of all positive results. The public sector played an important role in the diagnosis of these diseases through a contribution of 93% of all positive results. A strategy to improve completeness could be the active surveillance of the few public facilities with the highest numbers of positive results. Completeness of reporting could also be achieved by making it mandatory to report diagnoses from all laboratories.

For measles, the low number of cases diagnosed in laboratories and the high percentage of notifications from primary health centres implies the importance of clinical diagnosis and the lack of standard case definitions. Its reported rate is consistent with the incidences of measles in Turkey (8.1 per 100000) [19]. When incidence decreases with increasing control of disease, it becomes important to confirm suspected cases [22]. In former East Germany with very high vaccine coverage and incidence rates lowered to 0.7 per 100,000, 71% of notified cases were laboratory-confirmed compared with 34% in former West Germany with an incidence rate of 8.7 per 100,000 [23]. As Measles Elimination Programme has started in 2002 in Turkey, laboratory data can be expected to become more important in following years [24].

According to official incidences, Izmir could be classified as a low endemicity zone like Europe for hepatitis A while with the addition of cases discovered in laboratories, its incidence occurrence level becomes intermediate, like Turkey in general [25].

This study required an enormous effort to collect data which will never be feasible for routine surveillance. Methods for capturing these data could be computer queries to capture positive cases among laboratory results [26,27], and/or active surveillance in the few large-scale facilities where most cases occur.

## Conclusions

This is the first study quantifying completeness of communicable disease surveillance in Turkey. A considerable underreporting of hepatitis, brucellosis and syphilis has been found while surveillance of HIV was satisfactory. Mandatory laboratory based surveillance of communicable diseases should be incorporated into the surveillance system. If this is not possible, active surveillance could be carried out in a few large-scale tertiary public laboratories to capture most of the cases. Further research is also required to investigate the false positive test results and reasons of under-notification by clinicians.

## Competing interests

The authors declare that they have no competing interests.

## Authors' contributions

RD designed the study, collected and entered the data, analyzed them and prepared the manuscript. RD prepared her specialization thesis in Public Health with the first 6 months' data of this study. AOK was her advisor, and he critically reviewed the text.

## Pre-publication history

The pre-publication history for this paper can be accessed here:

http://www.biomedcentral.com/1471-2458/10/71/prepub
